# Melodic Contour Identification Reflects the Cognitive Threshold of Aging

**DOI:** 10.3389/fnagi.2016.00134

**Published:** 2016-06-13

**Authors:** Eunju Jeong, Hokyoung Ryu

**Affiliations:** Department of Arts and Technology, Hanyang UniversitySeoul, South Korea

**Keywords:** contour identification task, melodic contours, cognitive decline, aging, dorsolateral prefrontal cortex, hemodynamic responses

## Abstract

Cognitive decline is a natural phenomenon of aging. Although there exists a consensus that sensitivity to acoustic features of music is associated with such decline, no solid evidence has yet shown that structural elements and contexts of music explain this loss of cognitive performance. This study examined the extent and the type of cognitive decline that is related to the contour identification task (CIT) using tones with different pitches (i.e., melodic contours). Both younger and older adult groups participated in the CIT given in three listening conditions (i.e., focused, selective, and alternating). Behavioral data (accuracy and response times) and hemodynamic reactions were measured using functional near-infrared spectroscopy (fNIRS). Our findings showed cognitive declines in the older adult group but with a subtle difference from the younger adult group. The accuracy of the melodic CITs given in the target-like distraction task (CIT2) was significantly lower than that in the environmental noise (CIT1) condition in the older adult group, indicating that CIT2 may be a benchmark test for age-specific cognitive decline. The fNIRS findings also agreed with this interpretation, revealing significant increases in oxygenated hemoglobin (oxyHb) concentration in the younger (*p* < 0.05 for Δpre - on task; *p* < 0.01 for Δon – post task) rather than the older adult group (*n.s* for Δpre - on task; *n.s* for Δon – post task). We further concluded that the oxyHb difference was present in the brain regions near the right dorsolateral prefrontal cortex. Taken together, these findings suggest that CIT2 (i.e., the melodic contour task in the target-like distraction) is an optimized task that could indicate the degree and type of age-related cognitive decline.

## Introduction

Aging is believed to shift our attentional frame of reference more inwardly. Both seeing and hearing respond to such changes (Faubert, 2002; Parbery-Clark et al., 2011), and a certain level of withdrawal in cognitive functions is inevitable (Deary et al., 2009; De Oliveira et al., 2014). As the life expectancy increases, the loss of perceptual acuity and cognitive skills together cause significant difficulties in the elderly’s independence, such as communicating with others.

Speech in noise (SIN) perception, for example, requires an active interplay between auditory and cognitive capacities (Shinn-Cunningham and Best, 2008; Parbery-Clark et al., 2009a,b; Francis, 2010) and is one of the frequent difficulties that arises among older adults. When a speech stream is presented in a fairly quiet environment, attention to target speech is relatively easy. However, as the environmental noise, which contains meaningful sounds or utterances, becomes louder or competes against the speech stream. SIN perception therefore requires more effort and attentional resources (Pichora-Fuller, 2003, 2006; Heinrich et al., 2008; Russo and Pichora-Fuller, 2008). In other words, a higher level of auditory cognition, such as sound segregation and selective attention—the ability to separate important, relevant sound streams from extraneous ones—is necessary. In this respect, auditory scene analysis (ASA, Bregman, 1999) is likely intricately involved.

Many neurological studies suggest that older adults have a lower level of activation in the auditory cortex compared to younger adults (Ostroff et al., 2003; Snyder and Alain, 2005). In contrast, their cognitive-related cortices are highly activated. Notably, activation in cognitive areas other than the perceptual areas indicates an important compensatory mechanism for aging. This interpretation has been demonstrated in previous studies (e.g., Alain et al., 2009; Wong et al., 2009), which have reported that the elderly displays a more diffuse network involving the frontal and ventral brain regions, while younger people tend to show a more streamlined cortical network of auditory regions in response to spoken word processing in a noisy environment.

Taken together, these findings suggest an interactive contribution of the two factors in aging: the perceptual acuity of the target sound and the cognitive strategy to effectively cope with the target sound as well as the noise. Research, however, has failed to identify the contributions of each factor. Therefore, the present study presents the diverse acoustic features of music in an empirical study and examines the cognitive strategies and perceptual acuity used by both older and younger adults to cope with the exogenous complexity of the acoustic features of music (in particular, melodic contour) in this sense.

Music can be a methodologically time-efficient tool to evaluate cognitive changes that occur with aging. Notably, a pattern perception of music (i.e., melodic contour identification) often requires discrete pitch perception. Hearing loss and temporal resolution problems are generally observed in a pitch discrimination task (Tremblay et al., 2003), and an aging effect on cognitive functioning can also be seen in the neural substrates and their connectivity to musical stimuli (Bones and Plack, 2015).

**FIGURE 1:**
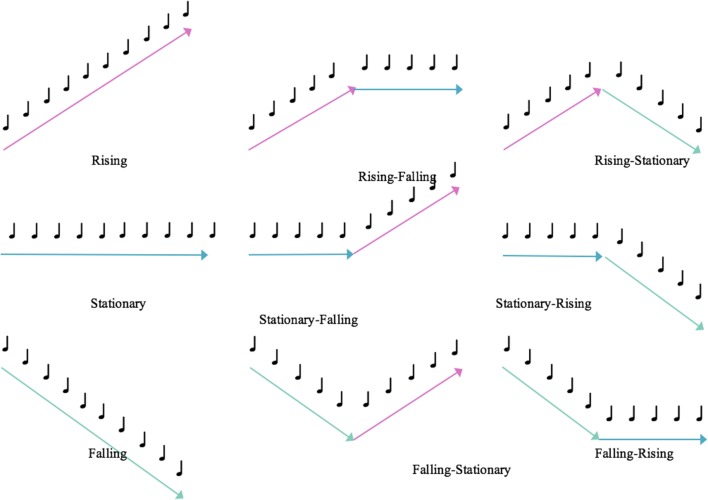
**Example patterns of melodic contour**.

Melodic contours share some common features with verbal speech and are the relative changes in pitch that are typically described as “rising,” “falling,” or “stationary.” As shown in **Figure 1**, different types of contours mimic prosodic information that can convey both non-verbal and subtle emotional aspects of speech (Inspector et al., 2013). In particular, previous researchers have found that contours and timbres are primarily processed at the brain stem level (Schirmer and Kotz, 2006; Leitman et al., 2009) and are the most noticeable compared to other structural elements of music across all age groups and cultures (Thompson and Schellenberg, 2006).

Previous studies have also suggested that melodic contours could be used as assessment stimuli for perceptual and cognitive dysfunctions. For example, a clinical application has been applied for cochlear implant (CI) problems (Galvin et al., 2008, 2009; Loui et al., 2008; Luo et al., 2014a,b). The general findings were that contour recognition and timbre perception are key in dictating auditory impairment. Even more interesting, Zhu et al. (2011) examined the effects of pitch, timbre, and time difference in the identification of simultaneous contours, and they reported that timbre and time differences were the most important features for the identification task. Therefore, these two features provide perceptual cues for an auditory stream and allow for segregation of the target auditory stimuli in a multi-layered auditory environment.

That being said, several empirical studies have used melodic contours to show cognitive decline in patient populations that have included those with traumatic brain injury and mild cognitive impairment (Belleville et al., 2011; Jeong and Lesiuk, 2011; Rahman et al., 2011; Jeong, 2013). Notably, a recent neurophysiological study (Jeong and Ryu, 2016) found that cognitive loads increase when distracting/competing sounds are present in the target sound identification task.

A note with regard to the melodic contour identification task (CIT) is further needed here. Both music and speech perception are types of time-based information processing and are subserved by the capacity of temporal processing and time resolution (Bones et al., 2014). In addition, up and down patterns in pitch contour are similar to the prosodies of human speech, which convey individuals’ emotional tones (Inspector et al., 2013).

These shared features between music and speech are processed in overlapping areas in the brain and in the medial prefrontal region in particular (Schirmer and Kotz, 2006; Patel, 2008; Fedorenko et al., 2009). This area modulates the junction between cognition and emotion and is inwardly directed toward mental states, including introspective thoughts about the self, autobiographical memory, and familiarity (Kelley et al., 2002; Platel et al., 2003; Gilbert et al., 2005; Ochsner and Gross, 2005), as well as tonal information of melodic contours (Platel et al., 2003; Janata, 2005; Wittfoth et al., 2010). The prefrontal region remains relatively intact until the final stage of dementia (Cuddy and Duffin, 2005), which suggests the possibility that it can be sensitive assessment stimuli to detect age-related cognitive impairments of aging.

Further, music processing assimilates real-world ASA. Generally, more than two streams (i.e., a melody and accompaniment or two melodies) are present in a musical scene. Likewise, in our auditory environment, multi-layered sound streams (e.g., voices, environmental sounds, and additional noises) are simultaneously present. The instrumental timbre provides a perceptual cue to segregate and/or integrate multi-layered streams of music, while voice timbre plays the same role in conversation against other environmental noises and/or among a crowd (Broadbent, 1958; Bregman, 1999). That is, one can employ diverse and subtle acoustic features, such as loudness, pitch, and timbre, to differentiate the target sound from the distracting sounds (Allen et al., 2011; Best et al., 2011; Luo et al., 2014a,b). Previous research (Janata, 2005; Jeong and Lesiuk, 2011) has claimed that, when given in a polyphonic texture, the melodic CIT can mimic the nature of real-world auditory surroundings in which environmental sounds, speech and music are simultaneously heard. For this study, the identification of the two melodic contours is thus considered as a representative experimental task.

It can be seen that timbre, in particular, is the primary cue for segregating concurrent melodic contours. The role of timbre in music scene analysis has also been well supported by neurological evidence (Satoh et al., 2001; Janata et al., 2002). In segregation, a salient or familiar timbre would be quickly processed, and the less salient or unfamiliar timbre might require a shift in attention. In this sense, selective attention is also examined. Hence, given that timbre discrimination is highly associated with speech perception in aging (Zhu et al., 2011; Grassi and Borella, 2013; Arehart et al., 2014), timbre discrimination and selective attention can sufficiently indicate age-related communication skills.

Taken together, these results indicate that the identification of melodic contour(s) given in a single- or multi-layered context represents one’s ability to segregate sounds and apply selective attention, while also imposing varying levels of cognitive load. The present study thus examines the behavioral and neurophysiological responses to the melodic CIT in various conditions and compares the difference between young and older adults in performance of the melodic CIT.

## Materials and Methods

### Participants

Thirteen college students (male = 10, female = 3) and 14 older adults (male = 7, female = 7) were voluntarily recruited via a web advertisement^1^. None of the participants were professionally trained in music and had a neurological medical history or sensory impairment. The mean age was 23.54 (*SD* = 1.66) years for the younger adults and 56.07 (*SD* = 6.35) years for the older adults. The average education level was 12.0 years for the younger adults and 12.43 years for the older adults with a non-significant difference between groups (*t* = -0.461, n.s.). All participants were right-handed, as indicated by the Edinburgh Handedness Inventory (Oldfield, 1971).

### Apparatus

#### Music Stimuli (Experimental Sound Sources)

**FIGURE 2:**
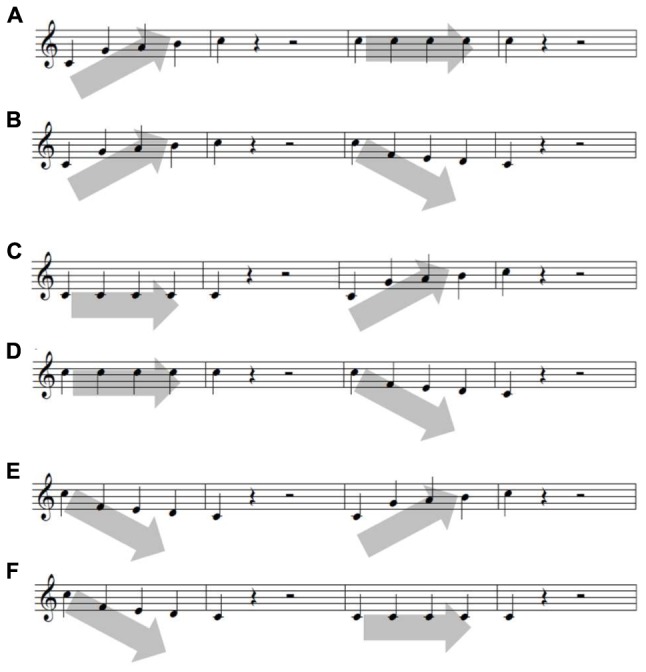
**Six sets of melodic contour**. Each of the six contours are **(A)** ascending-staying the same, **(B)** ascending–descending, **(C)** staying the same-ascending, **(D)** staying the same-descending, **(E)** descending–ascending, and **(F)** descending-staying the same.

The six contour stimuli were a combination of three contours (ascending, descending, and staying the same) adopted from Jeong (2013). Each contour consists of five tones presented at different frequencies as follows: (1) 262, 392, 440, 494, and 523 Hz for an ascending contour, (2) repetition of a single tone of 392 Hz for a staying the same contour, and (3) 523, 349, 330, 294, and 262 Hz for a descending contour. The contours were then combined in the following sets (**Figure 2**). The contours were played by one of three synthesized instruments (for timbre control: piano, flute, or string), and their amplitudes were identically normalized. The six contours in conjunction with the three instruments produced a total of 18 contour sets. All were generated using an MIDI synthesizer (YAMAHA DGX 230) connected to a Logic Pro X, and the experimental apparatus was implemented on a computer containing Visual Studio.

#### Contour Identification Task

**FIGURE 3:**
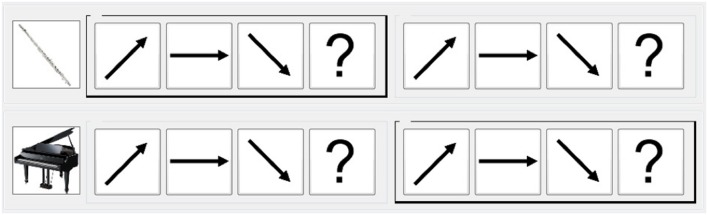
**Example of an answer sheet given in the CIT3**.

**Table 1** shows how the contours were combined in each of the three CITs. For CIT1, environmental sounds (e.g., raining, crying, laughing, babbling, and applause) were presented with the contours as a control condition. In contrast, in both CIT2 and CIT3, two different contour sets were presented at the same time using different instruments. In CIT2, participants were asked to selectively attend to a contour that was played by a specific instrument, which was cued on the computer monitor. For instance, two musical contours were played simultaneously by both the piano and the flute, and participants had to selectively attend to the flute as the target contour (**Figure 3**). CIT3 was more complex, and the participants were asked to shift their attention from one contour to another as the instruments displayed on the computer screen were changed. For example, the two sound contours were simultaneously presented by both the piano and the flute, and the indicator on the computer screen was changed from a piano (on the first contour) to a flute (for the second contour); participants were asked to separately identify the contour created by each instrument. For all CITs, the participants chosed the contour by clicking the arrows on the computer monitor (**Figure 3**).

**Table 1:** Structure of melodic contour identification task.

	Type of listening tasks		
Sound presented	Focused	Selective	Alternating
Melodic contour with	CIT1	–	–
environmental sounds			
Melodic contour with	–	CIT2	CIT3
target-like distraction			

### Measures

#### Hemodynamic Responses

**FIGURE 4:**
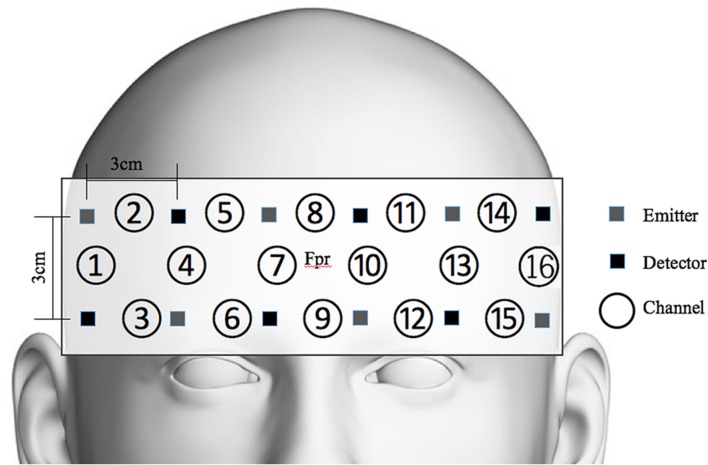
**Spectratech OEG-16**. The center of the measurement unit was placed on the frontopolar region (Fpr) according to the international 10–20 system.

OxyHb was measured to evaluate the participants’ cognitive activation and loads imposed by the given tasks (Peck et al., 2013; Sato et al., 2013; Ogawa et al., 2014; Yasumura et al., 2014). For this evaluation, functional near-infrared spectroscopy (fNIRS) and non-invasive monitoring of cortical tissue oxygenation (changes in both oxyHb and deoxygenated hemoglobin, HHb) during cognitive, motor, and sensory stimulation (Jobsis, 1977; Ferrari and Quaresima, 2012) were employed. We used a 16-channel Spectratech OEG-16 (Shimadzu Co. Ltd., Kyoto, Japan) (**Figure 4**). The task-related hemodynamic changes in amount of oxyHb were recorded in 16 channels with a sampling rate of 0.65 s. In addition to the fNIRS data, we collected behavioral data, including task performance accuracy and reaction time.

### Procedure

This study was approved by the Institutional Review Board of Hanyang University (HYI-14-127-3). Participants were voluntarily recruited via a web advertisement^1^. Prior to the experiment, all participants gave written informed consent in accordance with the Declaration of Helsinki.

On the day of the experiment, participants were administered the study instructions, and completed a demographic questionnaire. Then, a band-type NIRS containing an array of 12 probes was attached to each participant’s forehead. The probes were connected to the main board of the NIRS, which communicated with a computer. The melodic contour tasks were delivered via headphones with volume control; visual cues specifying the target musical stimulus were presented to the participants on a monitor (LG LED 24MA53D). A 20-s baseline was presented before, within, and after three CITs while the participants fixed their eyes on the center of the monitor. Prior to the main experimental sessions, the participants had practiced demonstrated a greater than 80% accuracy in the CIT.

Each CIT began with approximately 15 seconds of instructions, followed by the 18 items. The given CIT task was to identify the directions of the target contour by clicking the arrows shown on the screen after the melodic contour was presented. All of the items were programmed using Visual Studio. The tasks were given in an order of task complexity (i.e., CIT1 to CIT3) and items were presented in a random order within each of the three CITs. The participants’ behavioral and hemodynamic responses were recorded throughout the experiment. The CITs took about 20 min to complete. The experiment was performed in a sound-proof and light- and temperature-controlled room.

### Signal Processing and Statistical Analysis

The fNIRS raw data were collected and converted into concentrated changes of hemoglobin using the modified Beer–Lambert law. Then, a zero-phase low- and high-pass filter with a cutoff frequency of 0.01 to 0.09 Hz was applied using MATLAB (Morren et al., 2004; Akgul et al., 2005; Bauernfeind et al., 2011). Preprocessed oxyHb, which is known to be a sensitive indicator of cognitive load (Strangman et al., 2002; Holper and Wolf, 2011; Fishburn et al., 2014; Hwang et al., 2014; McKendrick et al., 2014), was averaged for pre-task, on-task, and post-task sessions. On-task sessions included three CITs, so the mean of oxyHb in each of the three CITs was estimated for statistical analyses.

A two-way mixed subject design was employed. The independent variables were age group (i.e., young vs. older), CIT, and session (pre-, on-, post tasks, for only NIRS data analysis). The dependent variables analyzed in this study were behavioral responses (performance accuracy, reaction time) and hemodynamic responses. We selected the following NIRS channels: channels 1 and 2 for right dorsolateral prefrontal cortex (DLPFC) activation and channels 14 and 15 for left DLPFC activation, which are known as sensitive regions to detect cognitive loads (Yasumura et al., 2014; Moriguchi et al., 2015). All statistical analyses were performed using Statistical Package for the Social Sciences (SPSS) ver. 20.

## Results

### Accuracy and Response Time

**FIGURE 5:**
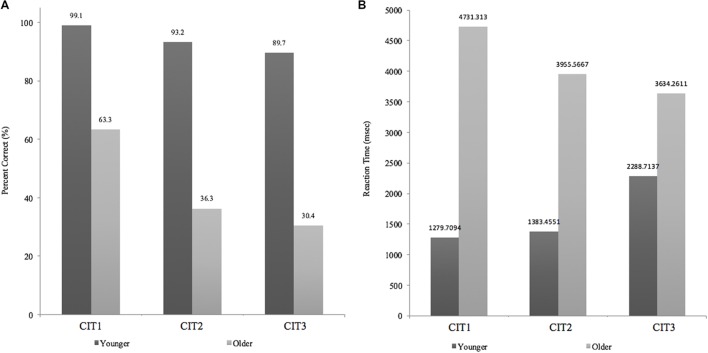
**(A)** Accuracy changes across CITs **(B)** Reaction times across CITs.

**Figure 5** shows the mean performance of the CIT with regard to accuracy (left panel, **Figure 5A**) and reaction time (right panel, **Figure 5B**) between the younger and older adult groups. The mean accuracy in the younger adult group was almost perfect for CIT1 given with the environmental sounds (99.1%), followed by CIT2 given with a melodic distractor (93.2%). Accuracy was the lowest (89.7%) when a shift between two concurrent melodic contours was required (i.e., CIT3). A similar trend across the CITs was found for the older adult group (ranging from 32.1–67.1%), but performance quickly decreased when performing both CIT2 and CIT3.

The percentage of correct responses was analyzed using group (young vs. older) x ΔCIT (ΔCIT1–CIT2, ΔCIT2–CIT3) two-way mixed analysis of variance (ANOVA). ΔCITs were considered to represent extra cognitive loads corresponding to the changes in task difficulty among CITs, which might indicate how the two different age groups managed the three CITs. There was a significant main effect of group (*F*_1,25_ = 15.351, *p* < 0.01) and ΔCIT (*F*_2,50_ = 14.273, *p* < 0.01), indicating that the overall performance of the young adult group was better than that of the older adult group and also that ΔCIT1–CIT2 was greater than ΔCIT2–CIT3. In addition, we found a significant group × ΔCIT two-way interaction (*F*_1,25_ = 9.262, *p* < 0.01). Pairwise *post hoc* analysis of each ΔCIT (Bonferroni–Dunn’s multiple comparison test) revealed a group difference at ΔCIT1–CIT2 (*p* < 0.001), indicating that the performance gap between CIT1 and CIT2, as compared to those between CIT2 and CIT3, was significantly higher for the older adult group (*p* < 0.001), while this was not the case for the younger adult group (*p* > 0.05, n.s).

**Figure 5B** presents the reaction times between the two age groups, which showed opposite patterns but, either difference between groups or ΔCIT1–CIT2 and ΔCIT2–CIT3 were not significant. Reaction time was further analyzed using a group × ΔCIT two-way mixed ANOVA. There was a significant main effect of group (*F*_1,25_ = 6.707, *p* < 0.05) but no significant main effect of ΔCIT (*F*_1,25_ = 4.001, *p* > 0.05) or an interaction effect (*F*_1,25_ = 1.375, *p* > 0.05), indicating that older adults required more time to complete any identification task than did younger adults.

Taken together, these results show that both accuracy and reaction time suggest a clear distinction between the young and older adults. The significant interaction effect observed between ΔCIT1–CIT2 and group with regard to accuracy implies that CIT2 (i.e., melodic contour identification with a target-like distraction) is sensitive enough to determine the difference between the young and the older age groups and those among three CITs in the older adult group.

### Hemodynamic Responses

Hemodynamic responses were analyzed using oxyHb obtained from channels 1 and 2 for the right DLPFC and 14 and 15 for the left DLPFC. OxyHb was analyzed across pre-task (baseline), on-task (performing task), and post-task (baseline) sessions. The oxyHb at on-task was sectioned into three parts to examine the changes according to CIT. **Table 2** presents the oxyHb changes across pre-, on-, and post-task sessions in the two different age groups. In general, oxyHb was increased compared to baseline after performing a task against baselines (i.e., pre-task and post-task), and this tendency differed by group.

**Table 2:** Descriptive statistics of oxyHb concentration.

Session	CIT	Channel	Group			
			Younger		Older	
			*M*	*SD*	*M*	*SD*
**Pre-task**		1	-0.0383	0.0440	- 0.0102	0.0095
		2	-0.0298	0.0546	-0.0070	0.0515
		14	-0.0241	0.0378	- 0.0117	0.0423
		15	-0.0260	0.0712	0.0069	0.0642
		
		*Average*	-0.02955	0.0519	- 0.0055	0.0419

**On-task**	CIT1	1	0.0037	0.0088	0.0007	0.0041
		2	0.0031	0.0039	- 0.0002	0.0029
		14	0.0015	0.0054	0.0001	0.0021
		15	0.0007	0.0054	- 0.0005	0.0037
		
		*Average*	0.0023	0.0059	0.0000	0.0032
	
	CIT2	1	0.0007	0.0058	0.0002	0.0017
		2	-0.0009	0.0048	- 0.0000	0.0020
		14	-0.0026	0.0047	0.0002	0.0032
		15	-0.0031	0.0058	0.0006	0.0042
		
		*Average*	-0.0015	0.0053	0.0003	0.0028
	
	CIT3	1	0.0037	0.0088	0.0008	0.0032
		2	0.0031	0.0039	0.0000	0.0027
		14	0.0015	0.0054	-0.0006	0.0021
		15	0.0007	0.0054	0.0010	0.0031
		
		*Average*	0.0023	0.0059	0.0003	0.0028

**Post-task**		1	-0.0472	0.1171	-0.0035	0.0292
		2	0.0203	0.0602	0.0053	0.0513
		14	0.0138	0.0556	0.0046	0.0410
		15	-0.0308	0.1210	- 0.0064	0.0293
		
		*Average*	-0.0110	0.0885	0	0.0377

The mean changes in oxyHb were analyzed using a group (young vs. older adults) × Δsession (Δpre – on task, Δon – post task) × channel (CH 1, 2, 14, 15) mixed ANOVA. There was a significant main effect of Δsession (*F*_1,25_ = 7.910, *p* < 0.01), but the group and channel effects were not significant (*F*_1,25_ = 0.356, *p* > 0.05; *F*_3,75_ = 1.773, *p* > 0.05, respectively). Interestingly, a significant interaction effect existed between group and Δsession (*F*_1,25_ = 4.401, *p* < 0.05). A *post hoc* pairwise comparison revealed that both the Δpre – on task and the Δon – post task were significantly different only for the young adult group (*p* < 0.05, *p* < 0.01, respectively). These findings indicate that the sensitivity and flexibility of oxyHb concentration in response to a stimulus were greater in the young adult group than they were in the older adult group.

**FIGURE 6:**
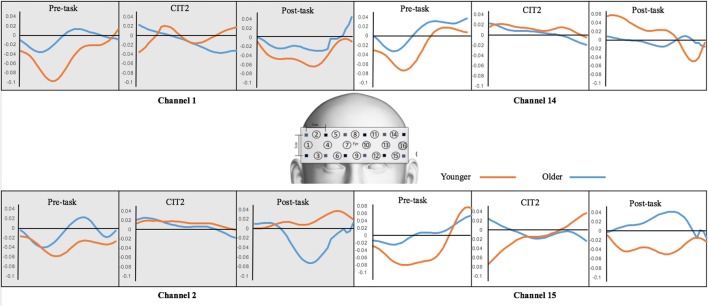
**Changes in oxyHb concentration**. OxyHb obtained from the pre-task, CIT2, and post-task were compared between the younger and older adult groups.

In the analysis of performance accuracy and response time, we found that CIT2 was of vital importance for determining the difference between the young and the older age groups and can be used as a critical task to represent cognitive functioning in the elderly. Therefore, we again compared the changes in oxyHb concentration between pre-task and CIT2 (ΔPre- CIT2) in order to determine if fNIRS data were consistent with the difference in behavioral performance (**Figure 6**).

The mean changes in ΔPre-CIT2 oxyHb were analyzed using a group × channel (1, 2, 14, 15) mixed ANOVA. There was a significant main effect of group (*F*_1,25_ = 4.882, *p* < 0.05). Further, we performed separate independent sample *t*-tests at each channel. ΔPre-CIT2 oxyHb on Channel 1 was greater in the young adult group than in the older adult group [*t*(25) = -2.336, *p* < 0.05]. These results suggest that the group difference at ΔPre-CIT2 was mostly due to the oxyHb changes that were increased in the right DLPFC in the younger adult group.

The changes in oxyHb concentration indicated that the young adults were more sensitive (as shown in ΔPre-on task and ΔPre-CIT2) and flexible (ΔOn-Post task) at performing melodic CITs. This tendency was in agreement with the participants’ behavioral performances (i.e., their accuracy and response times). In effect, CIT2 is a promising task to determine the difference between younger and older age groups and that within an older adults group (e.g., some elderly people may cope well with CIT2). The right DLPFC was a prominent brain area for detecting such a tendency due to the main characteristics of the given tasks. (Note that CITs are melodic CITs in which spatial cognition is vital. Also, the DLPFC is involved in complex cognitive functions such as executive functioning.)

## Discussion

The present study provides behavioral and neurophysiological evidence of the differences in melodic contour identification performance between younger and older adults. The behavioral results revealed that the cognitive declines observed in the older age group were obvious when participants were faced with a target surrounded by competing distraction (i.e., CIT2). Consistently, our fNIRS data showed a higher level of activation to the CITs in the younger age group than in older adults. The oxyHb concentration changes between the two groups were also greater when processing CIT2 at the right DLPFC (Channel 1).

### Behavioral Results

Overall, CITs using melodic contours in this study were shown to adequately indicate age-related cognitive declines. In particular, CIT2 (i.e., a selective listening task to target contours against target-like distractors) was considered a potential testing task to indicate a certain type of cognitive decline that typically occurs with aging. In general, accuracy gradually decreased across CITs and was lower in the older than in the younger adults. This finding is similar to those of previous studies that have reported that melodic contour identification performance decreased with aging (Mukari et al., 2010). This tendency is also consistent with previous music perception studies that have reported that such changes in music are probably due to decreased capacity in temporal and spatial resolution (Tremblay et al., 2003; Parbery-Clark et al., 2011). Neurological evidence has further indicated that the changes in connectivity and thickness of the insular cortex with aging (Heuninckx et al., 2005; Wong et al., 2009; Churchwell and Yurgelyn-Todd, 2013) might lead to decreased CIT performance.

One remarkable finding in this study is that a group difference was noted between CIT1 and CIT2, rather than CIT2 and CIT3. It is generally assumed that mental flexibility or shifting is more difficult than selective attention. However, changes between these two cognitive functions were not obvious in the older adult group, indicating when selective attention is intact or less affected, alternating attention is used for an indicator of cognitive evaluation. When it is affected, evaluation of the more complex cognitive function is meaningless –since the functions are posited in a hierarchy and if a fundamental function is not working, a higher one consequently cannot work.

Further, this tendency was more prominent in the older than in the younger adult group. These findings were possibly due to a reduced ability for timbre discrimination. Timbre is one of the key factors of ASA, as it provides a perceptual clue to how one organizes perceived auditory surroundings (Bregman, 1999; Neuhoff, 2011). For example, when two people speak simultaneously, we can typically distinguish between them based on voice timbre. Such timbre cues along with other pitch and loudness information, aid parsing, and tracking allow for distinction of the target voice in a complex auditory environment. This interpretation is very similar to those of previous studies that reported that listening to multilayered sounds, as an example of a cocktail party effect, is quite challenging with aging (Heine and Browning, 2002; Parbery-Clark et al., 2009b, 2011). A person’s ASA capacity rapidly decreases with aging, leading to loss of speech comprehension (Grassi and Borella, 2013; Arehart et al., 2014).

Higher-order cognitive processes, such as attention, stored knowledge, and goals, operate in ASA in concert with timbre discrimination (Alain et al., 2001). As described in Russo and Pichora-Fuller (2008), younger adults were more flexible in their ability to simultaneously zoom in and out to attend target and non-target sounds. Speech studies in the noise perception literature are also in line with the music studies mentioned above, indicating that the poorer performance of older adults has been attributed to declines in both hearing sensitivity (Russo and Pichora-Fuller, 2008; Parbery-Clark et al., 2011; Vermeire et al., 2015) and cognitive function (Guerreiro et al., 2010).

### fNIRS Results

Our findings were novel in that oxyHb activation showed music-specific PFC activation. Across sessions (pre-task, CITs, post-task), the oxyHb concentration summed from all four channels increased with CIT performance and returned to baseline at CIT completion. The discrepancies between baseline and CITs and between CITs back to baseline were much greater with younger than older adults. Also, as expected, the facilitating effect of music on CIT performance resulted in a higher oxyHb concentration in the right DLPFC. In other words, the oxyHb concentration in the right DLPFC greatly increased during CIT performance (i.e., ΔPre- CIT2), specifically for the younger adults. The current findings are indicative of changes in degree and type of cognitive capacity with aging.

The oxyHb increases in the right DLPFC between Pre-task and CIT2 are reflective of the essential characteristics of CITs. That is, the CITs in this study consisted of melodic contours, so the participants listened repeatedly to short melodies. The higher level of activation that was observed in the right hemisphere (RH) is in line with previous neuroimaging studies in which pitch pattern perception activated cognitive resources in the RH in an automatic and controlled manner (Harrington, 1987; Trainor et al., 2002; Zatorre et al., 2002; Lee et al., 2011). The PFC lateralization specific to task types (verbal recognition, Cabeza et al., 2003) and stuttering types (Kazenski, 2015) has been studied using fNIRS, but no investigations have focused on music processing. Hence, our findings firstly suggest right lateralization of the PFC in processing melodic contours provided in the selective listening task. More specifically, the higher level of right DLPFC enhancement between pre-task and CIT2 that was observed only in younger adults was in agreement with previous fNIRS studies that have revealed that older adults show less hemispheric lateralization in given cognitive tasks compared to younger adults (Tsjuii et al., 2010).

Second, our fNIRS data indicate that oxyHb concentration was more sensitive, ready for activation, and flexible to return to the neutral state in the younger than older adults. This finding is in agreement with previous fNIRS studies that claimed that older adults achieve a lower plateau of oxyHb regardless of any increase in task complexity, while younger adults tend to flexibly cope with task complexity increases (Mattay et al., 2006; Missonnier et al., 2011; Nagel et al., 2011). Other fNIRS studies have also supported our findings that younger adults showed higher activation in the PFC oxyHb concentration when performing more complex cognitive tasks compared to older adults (Herrmann et al., 2006; Vermeij et al., 2012).

These results can be further interpreted with regard to the roles of DLPFC. It is known that the DLPFC is activated by additional cognitive load so it can effectively modulate executive control and working memory (Opitz et al., 2002, 2005; Gaab et al., 2003; Barbey et al., 2013). In auditory modalities, the DLPFC is also consistently activated as the cognitive load increases (Alain et al., 2001; Seydell-Greenwald et al., 2013; Morita et al., 2015). In this sense, Lipschutz et al. (2002) claimed that greater DLPFC activation would be expected when performing a more complex cognitive task. Therefore, the changes in oxyHb concentrations that were noted in the right DLPFC (i.e., Channel 1) reflect both stimuli and task characteristics of the CITs.

### Conclusion and Future Suggestions

In conclusion, our findings showed that CIT performance could reflect the cognitive differences that exist between age groups. Note that younger adults performed well in the shifting tasks (i.e., CIT3); however, older adults showed a significant decrease in the selective attention task performed in conjunction with competing distractors (i.e., CIT2). Therefore, our findings imply that the detrimental cognitive functioning that occurs with aging can be effectively detected by the selective attention task rather than by shifting attention (i.e., flexibility) in music CITs. Also, our fNIRS data suggest that this tendency can be easily detected in the right DLPFC, indicating that declines in selective auditory attention and speech-in-noise perception are possibly associated with this region.

We recognize some limitations of the present study. The fNIRS was devised to observe PFC activation alone, so any activations in other brain regions and also the relationships between these regions and the PFC have not been accounted for; we therefore cannot determine whether cognitive declines that are associated with aging are solely dependent on the role of the PFC. Instead, this study focused on a non-verbal standardized evaluation task (note that musical non-verbal information processing is expected to be less knowledge- and culture-dependent), which empirically tests the validity of the CITs. An urgent future study would thus be a scaled-up experiment to examine the relationship between music and cognitive function in real-world situations. Another type of future study might be the use of CITs in patients with cognitive impairment (e.g., dementia, mild cognitive impairment). The end goal is to develop an early screening test for age-related cognitive functioning after determining how melodic contour can be efficiently employed in the clinical setting.

## Author Contributions

HR: Substantial contributions to the conception of the work; Revising it critically for important intellectual content; The interpretation of data for the work; Final approval of the version to be published; Agreement to be accountable for all aspects of the work in ensuring that questions related to the accuracy or integrity of any part of the work are appropriately investigated and resolved. EJ: Substantial contributions to the design of the work; The acquisition, analysis, and interpretation of data for the work; Drafting and revising the manuscript; Final approval of the version to be published; Agreement to be accountable for all aspects of the work in ensuring that questions related to the accuracy or integrity of any part of the work are appropriately investigated and resolved.

## Conflict of Interest Statement

The authors declare that the research was conducted in the absence of any commercial or financial relationships that could be construed as a potential conflict of interest.

## Footnotes

1 http://rise.hanyang.ac.kr.
